# 538. A Comparison of Self-reported to Provider Reported Fitzpatrick Skin Type in Pediatric Burn Patients

**DOI:** 10.1093/jbcr/irag033.235

**Published:** 2026-04-06

**Authors:** Chinaemelum C Akpunonu, Kara Paulk, Renata Fabia, Rajan K Thakkar, Dana M Schwartz

**Affiliations:** The Ohio State University Wexner Medical Center, Columbus, OH; Nationwide Children's Hospital, Columbus, OH; Nationwide Children's Hospital, Columbus, OH; Nationwide Children's Hospital, Columbus, OH; Nationwide Children's Hospital, Columbus, OH

## Abstract

**Introduction:**

The Fitzpatrick Skin Type (FST) is a numerical classification schema for skin color and its reaction to sunlight, used by providers to predict healing patterns and guide scar management. Similar to race categorization, studies have shown inaccuracies and discrepancies in self-reported vs patient-reported FST. Our pediatric burn clinic implemented a questionnaire for new patients to collect guardians’ assessment of their child’s FST, using a pictorial and descriptive chart. Experienced burn physicians used the same chart to assign patients an FST. We aimed to analyze the differences between physician vs self-reported FST at our institution.

**Methods:**

A retrospective review of burn patients aged 0-18 years seen at our ABA-verified Pediatric Burn Clinic from June 2024 to April 2025 was conducted. We collected patient and provider-reported FST scores and calculated the difference between those scores for each FST type using the Kruskal-Wallis test. We then performed an ordinal logistical analysis looking at age, sex, race, ethnicity, and language, to determine if these demographic factors influenced degree of differences between self-reported vs provider FST.

**Results:**

During the study, 228 unique patients were seen, with 96 patients having both self and physician-reported FST. Eleven patients (11.5%) self-identified as FST type 1, 31(32.3%) identified as FST type 2, 27 (28.1%) as FST type 3, 14 (14.6%) FST type 4, 10 (10.4%) as FST type 5 and 3 (3.1%) as FST type 6. Comparing physician-reported to self-reported FST for each type, the FST 1 group had a median difference of 0 [IQR 0,0]. FST 2 group had a median difference of –1 [IQR (-1), -1], FST 3 had median of –1 [IQR (-2), 0], FST 4 group had median of 1 [IQR 0,1], FST 5 had a median of 0.5 [IQR 0, 1], and FST 6 had a median difference of 0 [IQR 0,0]. *p* value for comparing the difference between provider and patient FST for all FST was *p*<.0001. Comparing patient demographic factors predictive of difference between physician and self-reported FST, race significantly affected the likelihood of FST underestimation (OR 2.03 (CI 1.3-3.2, *p*=.002). Compared to white patients, black, Asian, and multiracial patients had an increased risk of having their FST underestimated with odds ratios of 76.96 (CI 20.9-283.9), 12.5 (CI 1.6-96.2), and 10.0 (CI 2.6-38.7) respectively.

**Conclusions:**

Physicians tended to assess FST more concordantly with patients at the lowest and highest FST groups (1 and 6) but had a greater median difference in scores for the middle FST groups (2-5). Providers tended to assign a lower FST type than patients assign themselves. Nonwhite patients had a higher likelihood of physicians’ underscoring their FST.

**Applicability of Research to Practice:**

Most burn literature references FST as an objective demographic factor, however this data indicates variability between self-reported and physician-reported FST. Centers looking at effects of FST on burn healing and scar management should report the source of FST assignment.

**Funding for the study:**

N/A.

